# The mutation p.E113K in the Schiff base counterion of rhodopsin is associated with two distinct retinal phenotypes within the same family

**DOI:** 10.1038/srep36208

**Published:** 2016-11-04

**Authors:** Charlotte Reiff, Marta Owczarek-Lipska, Georg Spital, Carsten Röger, Hebke Hinz, Christoph Jüschke, Holger Thiele, Janine Altmüller, Peter Nürnberg, Romain Da Costa, John Neidhardt

**Affiliations:** 1 Eye Center, Albert-Ludwigs-University of Freiburg, Freiburg, Germany; 2 Human Genetics, Faculty of Medicine and Health Sciences, University of Oldenburg, Oldenburg, Germany; 3 Department of Ophthalmology, St. Franziskus-Hospital, Münster, Germany; 4 Cologne Center for Genomics, University of Cologne, Cologne, Germany

## Abstract

The diagnoses of retinitis pigmentosa (RP) and stationary night blindness (CSNB) are two distinct clinical entities belonging to a group of clinically and genetically heterogeneous retinal diseases. The current study focused on the identification of causative mutations in the RP-affected index patient and in several members of the same family that reported a phenotype resembling CSNB. Ophthalmological examinations of the index patient confirmed a typical form of RP. In contrast, clinical characterizations and ERGs of another affected family member showed the Riggs-type CSNB lacking signs of RP. Applying whole exome sequencing we detected the non-synonymous substitution c.337G > A, p.E113 K in the rhodopsin (*RHO*) gene. The mutation co-segregated with the diseases. The identification of the pathogenic variant p.E113 K is the first description of a naturally-occurring mutation in the Schiff base counterion of RHO in human patients. The heterozygous mutation c.337G > A in exon 1 was confirmed in the index patient as well as in five CSNB-affected relatives. This pathogenic sequence change was excluded in a healthy family member and in 199 ethnically matched controls. Our findings suggest that a mutation in the biochemically well-characterized counterion p.E113 in RHO can be associated with RP or Riggs-type CSNB, even within the same family.

Retinal dystrophies (RDs) represent a group of inherited disorders leading to dysfunction or death of retinal cells that might even progress towards complete loss of vision^1^. RDs might be classified, depending on their phenotypic presentation, into syndromic and non-syndromic forms^2^. In the syndromic forms of RDs the ocular phenotype is accompanied with pathogenic changes in other organs or tissues, whereas the phenotype in non-syndromic forms is limited to the retina. RDs are also categorized according to the type of affected retinal cells: rod photoreceptor dominated, cone photoreceptor dominated, generalized or macular retinal dystrophies, and vitreoretinopathies^2^.

Retinitis pigmentosa (RP) belongs to a group of clinically and genetically heterogeneous RDs and predominantly affects the function of rod photoreceptors, bipolar cells or the retinal pigment epithelium^3^,^4^. The frequency of RP is estimated to be between 1 in 3000 and 1 in 7000 individuals worldwide^5^. Characteristically, the retinal degeneration starts in the mid-periphery. It may progress and affect the macula and fovea. Disease symptoms frequently start early in life and progress with time. For most cases, the severe stage of the disease typically occurs between the third and fifth decade of life^5^,^6^. A continuing reduction of mid- and peripheral visual fields often leads to tunnel vision. In the course of the disease, cone photoreceptor loss may result in photophobia and even complete blindness^7^,^8^. Initially, RP-affected patients suffer from night blindness, due to a functional defect in rod photoreceptors or associated retinal neurons.

In contrast to RP, CSNB patients present a mostly non-progressive form of night blindness that lacks clear signs of retinal degeneration^4^. CSNB with normal fundi can be categorized according to findings in full field electroretinograms (ERG) into two types, Riggs^9^ and Schubert-Bornschein^10^. The Riggs-type is characterized by a proportional reduction of a- and b-waves^4^,^9^,^11^, whereas scotopic ERG findings in the Schubert-Bornschein type show normal a-wave amplitude and severely reduced b-wave amplitude^4^,^10^. The Schubert-Bornschein-type has been additionally classified into a complete and incomplete form, with ON-bipolar or ON- and OFF-bipolar dysfunctions, respectively^4^,^12^,^13^. Non-syndromic forms of RP and CSNB follow three different modes of inheritance: autosomal recessive, autosomal dominant and X-linked^4^,^14^. Until now, more than 60 RP genes and more than 10 CSNB genes have been identified^2^ (RetNet, https://sph.uth.edu/retnet/). They show expression patterns in rod photoreceptors and/or retinal pigment epithelium, are involved in the phototransduction pathway and retinal metabolism, or play a role in structure or maintenance of retinal cells^7^. Rhodopsin (*RHO*) has been associated with more than 180 mutations (http://www.biobase-international.com/product/hgmd). These mutations explain approximately 5–10% of all RP cases. Moreover, up to 30–40% of families with autosomal dominantly inherited RP carry disease-associated variants in *RHO*^3^,^5^,^15^. Until now four mutations (A292E, G90D, T94I, and A295V) in the *RHO* gene were reported to cause autosomal dominant CSNB^16^,^17^,^18^,^19^.

RHO is a seven transmembrane G-protein coupled receptor located in rod photoreceptors. It contains the chromophore 11-cis-retinal. This chromophore is covalently linked to the apoprotein opsin by the protonated Schiff base at Lys-296 and is stabilized by the counterion at p.Glu113 of RHO^20^. During light absorption the 11-cis-retinal chromophore isomerizes to the all-trans form. At this point RHO is activated and binds to the G-protein transducin (GNAT1), which in turn associates with the phosphodiesterase-6 (PDE6). The amount of cytoplasmic cyclic guanosine 3′,5′-monophophate (cGMP) is reduced due to the activation of PDE6. Consequently, cGMP-activated channels are closed in the plasma membrane of rod photoreceptors. In dark conditions the cGMP-activated channels are opened, thereby sodium and calcium ions enter outer segments of photoreceptors. Under light stimuli the cGMP channels close resulting in a decreased calcium concentration in photoreceptors^4^,^21^,^22^. A phosphorylation of RHO turns off the phototransduction pathway and leads to the exchange of all-trans retinal into 11-cis-retinal. This enables RHO to be activated again by light stimuli^23^,^24^. Various substitutions in the RHO Schiff base Lys-296 and at the RHO counterion Glu113 were characterized *in vitro* and strongly suggested the constitutive activation of the apoprotein^18^,^25^. Such constitutive activation leads to a lower light sensitivity of the phototransduction cascade, a likely explanation for the night blindness phenotype observed in patients.

Herein, we describe for the first time in human patients a single nucleotide substitution that occurred in the Schiff base counterion p.E113 of RHO. Surprisingly, the mutation can be associated with the two distinct phenotypes of RP and Riggs-type CSNB in independent members of the same family.

## Material and Methods

### Patients

The index patient and 6 additional members of a three-generation family were analyzed. All members originated from Germany, were informed in detail about the project and consequences of the study and formally agreed to participate in the study by informed written consent. The study adhered to the tenets of the Declaration of Helsinki and was approved by the local ethics committee (Hannover Medical School (MHH) ethics committee, OE9515).

### Ophthalmic investigations

The index patient (III-1) and 6 additional family members were queried about visual abilities including visual acuity, nyctalopia, visual fields, photophobia and color vision problems. A complete phenotype characterization of the index and family member II-3 was performed including best corrected visual acuity, slit lamp examination, funduscopy, Goldmann kinetic perimetry, fundus autofluorescence (FAF) of the central 30° recorded with a standard confocal scanning laser ophthalmoscope (Heidelberg Retina Angiograph [HRA]; Heidelberg Engineering, Heidelberg, Germany) and macular optical coherence tomography (Spectralis OCT, Heidelberg Engineering, Heidelberg, Germany). Binocular full-field ERGs (RP patient: maximum flash intensity 10 cd*s/m^2^; Nicolet, Madison, WI; CSNB patient: maximum flash intensity 15 cd*s/m^2^, Toennies/ Erich Jäger GmbH, Hoechberg, Germany and multifocal ERGs (mfERG; VERIS 4.8, Electro-Diagnostic Imaging, Redwood City, CA) for the RP patient were recorded according to ISCEV (International Society for Clinical Electrophysiology of Vision) standards^26^ and guidelines^27^. Fundi of the RP patient were documented with a model FF450 fundus camera (Carl Zeiss Meditec GmbH, Jena, Germany)^28^. The pedigree was assembled according to clinical examinations, interviews, and/or ophthalmological reports of 7 family members. Additional family members were not available for clinical analyses or interviews.

### DNA isolation

Peripheral blood samples from 6 affected and 1 non-affected family member and 199 control individuals were collected in EDTA tubes. DNA extraction was performed using the MagCore^®^ Super Nucleic Acid Extraction System (NIPPON Genetics EUROPE GmbH, Düren, Germany) or standard salting out procedures (Gentra Puregene Kit, QIAGEN GmbH, Hilden, Germany) according to manufacturer’s instructions.

### Whole exome sequencing

Whole exome sequencing of the genomic DNA extracted from the blood sample of the RP-index patient was performed at the Cologne Center for Genomics, University of Cologne (http://portal.ccg.uni-koeln.de/ccg/ngs/). The SeqCap EZ Human Exome Library v2.0 (Roche NimbelGen Inc., Madison, WI) was used to enrich the whole exome of the RP-affected patient and analysed with the paired-end 2 × 100 bp protocol and v3 chemistry on the Illumina HiSeq system. The sequence reads were mapped to the human reference genome (hg19) using the Burrows-Wheeler Aligner (BWA-aln)^29^. Variant calling and annotation was performed with Varbank (https://varbank.ccg.uni-koeln.de/) including Samtools^30^, GATK^31^ and Picard. High quality variant calls (following GATK best practice filtering)^32^ were accepted for further analyses after passing the following filtering: (a) predicted changes on protein structure or splicing alterations, (b) allele frequency in the Caucasian population ≤ 0.01, (c) allele frequency in a Varbank analysed European epilepsy cohort (n = 511) ≤ 0.02, (d) allelic reads to reference reads ratio > 0.25.

### PCR amplification and Sanger sequencing

Primers encompassing the entire coding region and splice sites of the *RHO* gene (Genbank: NG_009115.1, NM_000539.3) were designed using the Primer3 software^33^. Five PCR fragments from genomic DNA of the index patient and accessible family members were amplified using HotFirePol DNA Polymerase (Solis BioDyne, Tartu, Estonia) and primers pairs: Ex1-fwd 5′_AGCTCAGGCCTTCGCAGCAT, Ex1-rev 5′_GAGGGCTTTGGATAACATTG; Ex2-fwd 5′_GAGTGCACCCTCCTTAGGCA, Ex2-rev 5′_TCCTGACTGGAGGAC; Ex3-fwd 5′_CTGTTCCCAAGTCCCTCACA, Ex3-rev 5′_CTGGACCCTCAGAGCCGTGA; Ex4-fwd 5′_CAGCATGCATCTGCGGCTC, Ex4-rev 5′_CCTGGGAAGTAGCTTGTCCTT; and Ex5-fwd 5′_GCCAGTTCCAAGCACACTGT, Ex5-rev 5′_GACTTCGTTCATTCTGCACAG, respectively^3^. Additionally, 398 control alleles were screened with primers spanning exon 1 of *RHO*. All PCR reactions were performed using the following thermal conditions: initial denaturation at 95 °C for 15 minutes, denaturation at 95 °C for 45 seconds, annealing at 60 °C for 45 seconds, elongation at 72 °C for 30 seconds for 35 cycles, and final elongation at 72 °C for 10 minutes. The amplicons were purified with exonuclease I and shrimp alkaline phosphatase (New England Biolabs, Frankfurt, Germany) and directly sequenced using the BigDye^®^ Terminator v3.1 Cycle Sequencing Kit and ABI Prism 3130xl Genetic Analyzer (Applied Biosystem, Carlsbad, California, USA). Sanger sequencing data were subsequently analysed with Sequence Scanner v.1.0 and SeqScape software (Applied Biosystem, Carlsbad, California, USA).

### Multiple sequence alignment of RHO protein

The amino acid sequences of the RHO protein were compared between human (Genbank: NP_000530.1), chimpanzee (Genbank: XP_516740.2), cattle (Genbank: NP_001014890.1), dog (Genbank: NP_001008277.1), cat (Genbank: NP_001009242.1), mouse (Genbank: NP_663358.1), opossum (Genbank: XP_001366225.1), chicken (Genbank: NP_001025777.1), African clawed frog (Genbank: NP_001080517.1) and zebrafish (Genbank: NP_571287.1) using the ClustalW2 multiple alignment program (http://www.ebi.ac.uk/Tools/msa/clustalw2/).

## Results

### Clinical characterizations

**Figure 1:**
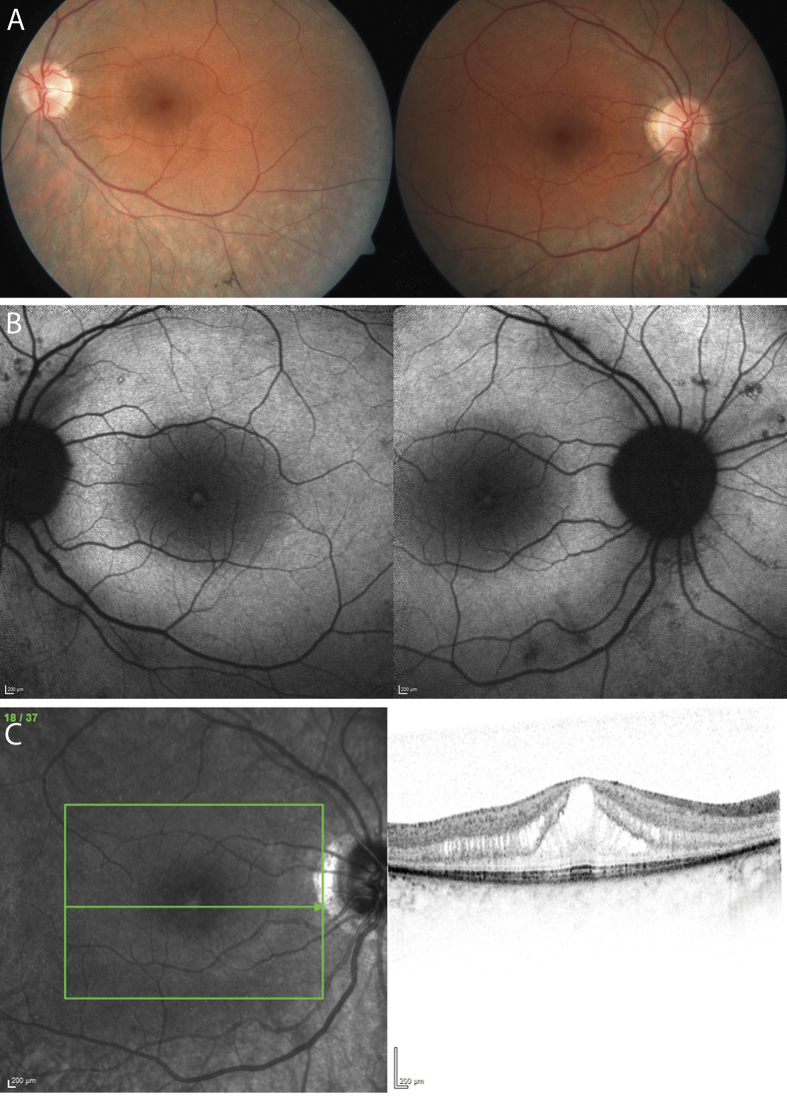
Clinical characterization of the index patient. Fundus photographs (**A**) and FAF recordings (**B**) of the index patient’s left and right eye are shown. Due to high interocular symmetry the right eye is not shown for OCT analyses (**C**). Fundi show midperipheral loss of choriocapillaris, constricted retinal vessels, isolated retinal vessel-associated pigmentary clumping and foveal thinning (FA). FAF demonstrates a ring-shaped perifoveal increase, sporadic granular decrease along the vascular arcades and a well-defined foveal increase (**B**) due to cystoid retinal changes as demonstrated by macular OCT (**C**).

The 33-year-old index patient noticed first signs of nyctalopia since childhood. For the past 2 years the patient described worsening nyctalopia, onset of photophobia, and worsening peripheral visual fields with subjective preservation of central visual acuity. Best corrected visual acuity was 20/32 (right eye) and 20/25 (left eye) in standard room illumination. Fundus examination revealed midperipheral loss of choriocapillaris, constricted retinal vessels, isolated retinal vessel-associated pigmentary clumping and foveal thinning (Fig. 1A). Goldmann perimetry revealed a typical midperipheral ring scotoma and concentric constriction of the remaining central 20° (data not shown). FAF showed a ring-shaped perifoveal increase, sporadic granular decrease along the vascular arcades and a well-defined foveal increase due to cystoid retinal changes as demonstrated by macular OCT (Fig. 1B,C). The dark-adapted ERG showed pathologically reduced, but detectable and reproducible minor amplitudes in both eyes. Due to severe amplitude reduction a reliable measurement of a- and b-wave peak times was not possible. A discrete Fourier transform of the flicker response revealed minor significant cone responses of the left eye only and significant peak time prolongation (see Supplementary Fig. S1; for comparison to the normal ERG recording see Supplementary Fig. S2). MfERG demonstrated a pathological central amplitude reduction and decreasing amplitudes with increasing eccentricity of the stimulus. Together, these findings showed a moderate manifestation of the RP phenotype in the index patient.

**Figure 2:**
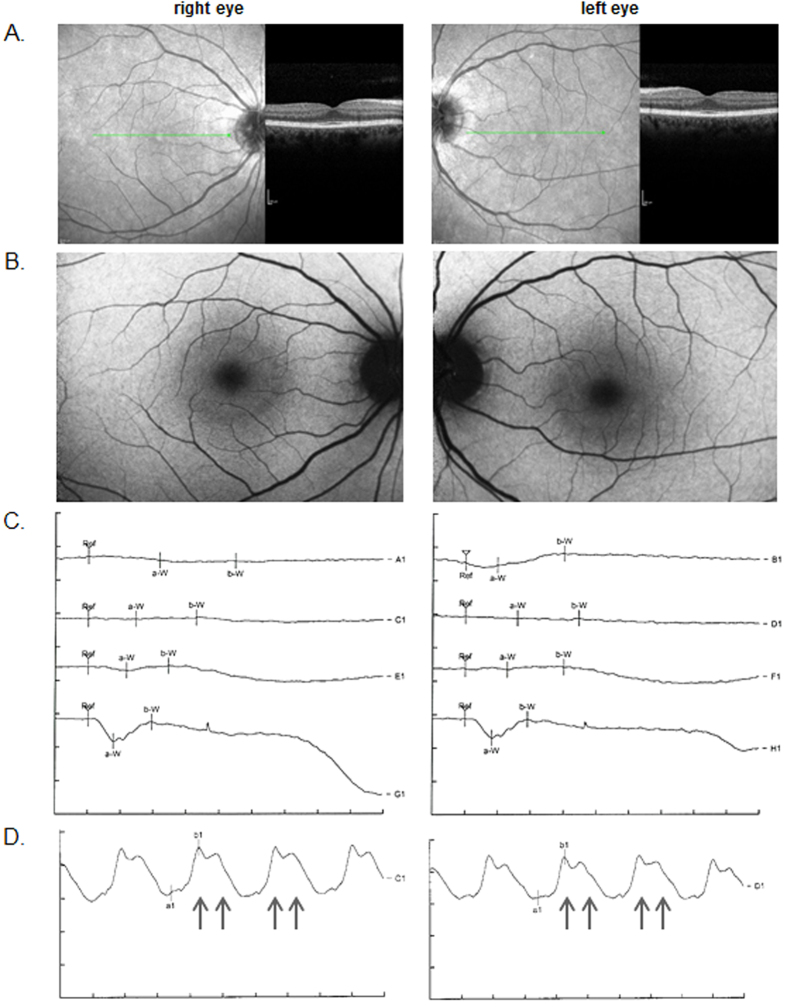
CSNB clinics. Central OCT scan (**A**), fundus autofluoresence (**B**) and ERG recordings (**C,D**) of both eyes are shown. The scotopic ERGs (**C**) were measured in both eyes with four intensities: 0.4 mcd*s/m^2^ (A1, B1), 4 mcd*s/m^2^ (C1, D1), 40 mcd*s/m^2^ (E1, F1), and 2.5 cd*s/m^2^ (G1, H1). Per division mark, units on the x and y axes show 20 ms and 100 μV, respectively. The flicker ERGs (**D**) with the 2.5 cd*s/m^2^ intensity are shown in the right and in left eye. The arrows point towards the bifid peaks in the flicker ERG. Units on the x and y axes display 15 ms and 40 μV per division mark, respectively.

In contrast, the 51-year-old patient II-3 reported a non-progressive nyctalopia from an early age, lacking any signs of photophobia, color vision defects, and reduced peripheral visual fields. Best corrected visual acuity was (20/20) for both eyes in standard room illumination. Anterior segments were normal with only very mild symmetric bilateral nuclear lens opacification. The posterior pole showed an unremarkable appearance on both sides. The left eye showed a few subtle RPE irregularities only in the temporal and inferior periphery (see Supplementary Fig. S3). There was no finding of peripheral vessel constriction or any “bone spicule” shaped pattern of fundus hyperpigmentation. Central FAF showed a subtle perifoveal ring-shaped increase (Fig. 2B). Peripheral FAF showed a few irregular reductions restricted to the far temporal and inferior periphery of the left eye. The macular OCT did not show cystic changes or other abnormalities in the outer or inner retinal layers. No reduction of retinal layer thickness was detected (Fig. 2A). The ERG recordings demonstrated a decreased a-wave in response to the scotopic bright flash and a reduction in the b/a-wave ratio resembling an electronegative ERG recording (Fig. 2C). This finding is in accordance with the Riggs-type CSNB^4^. The photopic ERG suggested slightly reduced, near to normal cone responses (see Supplementary Fig. S4). Surprisingly, the photopic 30 Hz flicker ERG clearly demonstrated a double peak (please compare Fig. 2 and Supplementary Fig. S5). ERG-waves of a healthy person are shown in Supplementary Figs S5 and S6.

Four other relatives (I-2, II-2, III-2, and III-6) were interviewed and occasionally provided reports from different ophthalmologists, but did not express their will to participate in an additional clinical investigation involving ERG recordings. The data from relatives: I-2, II-2, III-2, and III-6 supported the conclusion that they were only affected by CSNB and lacked any signs of RP. Even the oldest family member (I-2, 82 years old) showed no signs of myopia (both eyes 0.5 diopter) and no reduction of peripheral vision. Her fundus was judged to be normal by a local ophthalmologist. The ophthalmologist’s reports and interview did not reveal hints towards nystagmus, strabismus or photophobia. Thus, the phenotype of family members I-2, II-2, III-2, and III-6 seems to be in accordance with the clinically supported Riggs-type CSNB in the II-3 patient. These findings suggest that all affected family members manifested the Riggs-type CSNB with the exception of the index patient who was clearly diagnosed with RP.

### Whole exome sequencing analysis

**Figure 3:**
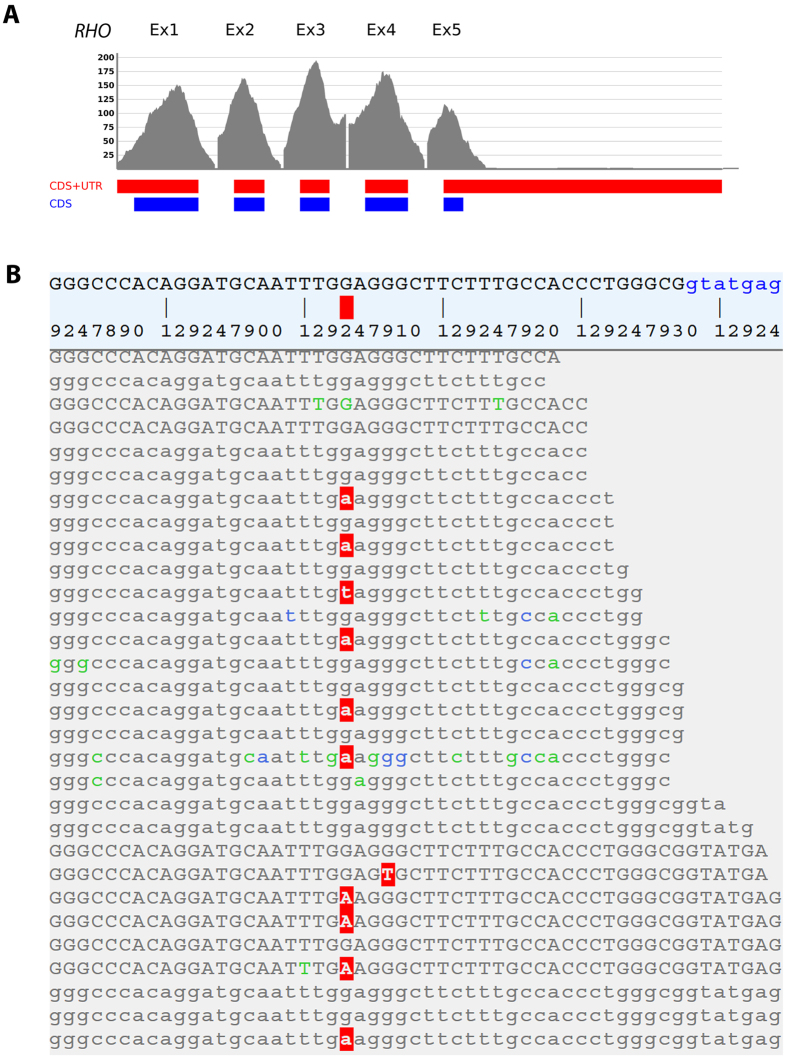
Schematic representation of coverage of sequencing reads in *RHO* gene. (**A**) Sequencing coverage is shown on the Y-axis. The X-axis schematically presents the five exons of *RHO*. CDS: coding sequence, UTR: untranslated regions. (**B**) The G to A nucleotide exchange in exon 1 of *RHO* was identified by next generation exome sequencing. A representative selection of the sequence alignment is shown. The identified c.337G > A mutation is indicated by the red square above the sequence. Blue-, green-, and grey-colored bases represent a phred-scaled base quality score of 0-9, 10-29, and over 29, respectively.

We applied whole exome sequencing analysis as an efficient method to screen for mutations in genetically heterogeneous disorders, such as RP or CSNB. Sequencing of the coding regions and flanking intronic parts of genomic DNA from the index patient resulted in a total of 6.6 Gb uniquely mapped bases, a 30x coverage of 89.5% and a mean coverage of 85 times. Genomic variants of the patient sample including single nucleotide variations (SNVs) and indel variants were identified compared to the human reference genome (see Supplementary Table S1). After filtering, the whole exome sequencing revealed 379 SNVs (including 6 splice sites, 347 non-synonymous, 9 nonsense, 3 synonymous) and 7 INDEL variants on target regions. Further filtering included prioritization of the variants and reduced the number of likely pathogenic sequence changes to 200. Out of these variants, only one mutation was located in a known candidate gene for autosomal dominant RP or CSNB. Thus, the non-synonymous substitution in *RHO* (NM_000539.3, c.337G > A, p.E113 K) was likely to be the disease-associated sequence alteration explaining the phenotypes found in the family described herein. The complete coding sequence (CDS) of *RHO* was analyzed with the mean coverages of 39x for exon 1, 62x for exon 2, 97x for exon 3, 73x for exon 4, and 52x for exon 5 (Fig. 3A). The identified heterozygous alteration G to A in the exon 1, at the nucleotide position 337 (c.337G > A) was sequenced with a total of 89 times coverage. The wild type G allele and the mutated A allele were present in 64% and 34% of the sequencing reads, respectively (Fig. 3B). The heterozygous *RHO* alteration resulted in an exchange of the glutamic acid (codon: GAG) at the protein position 113 into lysine (codon: AAG).

### Analysis of the *RHO* mutation

**Figure 4:**
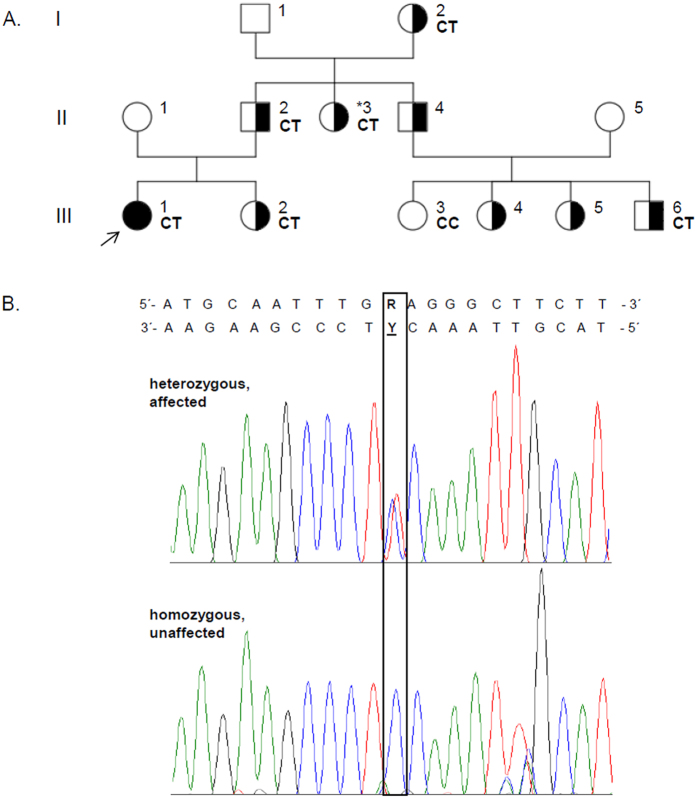
Genetic analysis of the family affected by RP. (**A**) The family pedigree shows three generations (I, II, and III) and 13 family members. The RP-affected individual is indicated by a solid black circle (additionally marked with an arrow). The family member affected by the clinically supported Riggs-type CSNB is shown as a half-filled symbol in combination with an asterisk, whereas those family members that show a presumed Riggs-type CSNB are shown with half-filled symbols only (boxes and circles). Among them, 7 relatives were screened for the c.337G > A mutation. The identified genotype is indicated on the right side of each analyzed individual. CT shows the presence of the heterozygous mutation, whereas CC indicates the presence of two control alleles. (**B**) Sequencing analysis of the c.337G > A mutation in exon 1 of the *RHO* gene. The chromatograms were obtained by reverse-direction sequencing of the genomic DNA from the index patient and from a healthy family member. The position of the identified mutation is marked by a black frame and it is specified by “R” or “Y”, on the forward strand (G to A exchange) or the reverse strand (C to T exchange), respectively.

The five exons of *RHO* were sequenced from both directions in the available family members in order to exclude any additional coding or exon flanking genetic variants in this gene. We confirmed the mutation c.337G > A, p.E113K in *RHO*. Sequencing of exon 1 in all available family members showed the co-segregation of the c.337G > A mutation with the phenotypes. The heterozygous mutation was found in the index patient diagnosed with RP (III-1; Fig. 4A,B) as well as in 5 additional family members with the assumed Riggs-type CSNB (I-2, II-2, II-3, III-2 and III-6; Fig. 4A). Family member III-3 was not affected and did not show the *RHO* mutation. Three of the affected family members (II-4, III-4, and III-5; Fig. 4A) did not agree to participate neither in the clinical investigations nor in the screening of the *RHO* c.337G > A mutation.

The *RHO* c.337G > A mutation was not identified in ~60000 exomes from the Exome Aggregation Consortium (ExAC, Cambridge, MA (URL: http://exac.broadinstitute.org) [05/2015]). In addition, genetic screening of 398 alleles from control individuals did not show a single case presenting with the *RHO* mutation. These data confirm the rare nature of the mutation that co-segregates with the disease in the family.

Three additional genetic variants in 5′UTR (c.−26A > G), intron 3 (c.696 + 4C > T), and in 3′UTR (c.*46C > A) of *RHO* were found in all examined family members. These sequence variants are likely to be non-pathologic as they frequently occur in general human populations (see Supplementary Table S2).

Only the index patient showed the phenotype of RP, whereas other family members showed the presumed Riggs-type CSNB. It seemed possible that the index patient carried additional sequence alterations that might explain the more severe RP phenotype. Using our whole exome sequencing data of the index, we searched for additional mutations in known genes of retinal degeneration. This analysis did not reveal any sequence alterations that would explain why the index patient is affected by RP rather than the Riggs-type CSNB. However, additional genetic alterations located in other genes, which have not yet been associated with RP as well as non-coding variants and epigenetic factors cannot be excluded.

### Multiple species comparison of RHO sequences

**Figure 5:**
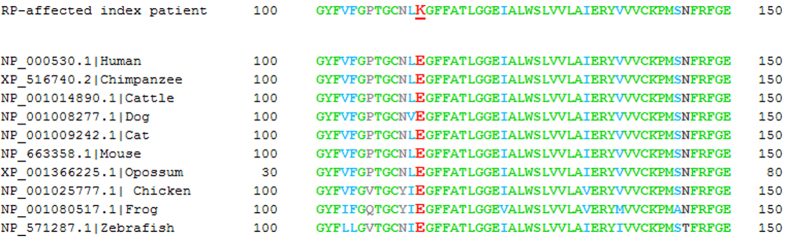
Multiple sequence alignment of RHO protein sequences. The aligned fragments include RHO-derived amino acids between positions 100 and 150, and encompasses the p.E113 counterion residue. The corresponding fragment of 50 amino acids from opossum starts at the position 30. The RHO mutation p.E113 K was identified in the RP-affected index patient and it is underlined and marked in red. The position p.E113 in the remaining protein sequences is also marked in red, whereas other identical residues, conserved substitutions, semi-conserved substitutions, and no matches are marked in green, blue, dark grey, and bright grey, respectively.

Multiple sequence alignment of RHO protein sequences showed uniformity of the glutamic acid residue 113 (p.E113). Full conservation of p.E113 was found across 10 divergent species. The altered RHO protein sequence found in the RP-affected index patient was also aligned to these orthologous sequences (Fig. 5). The exchange of p.E113 K alters a highly conserved sequence position in RHO and further supports the importance of this amino acid for structure and/or function of RHO. It has been reported that p.E113, together with p.K296, are essential for visual pigment positioning within RHO as well as its ability to trigger the phototransduction cascade^34^.

## Discussion

Next generation sequencing technology allowed us to efficiently sequence the whole exome of the index patient in a three-generation family affected with RP and CSNB. We identified a disease-associated mutation in RHO (p.E113 K). The segregation of the phenotype within the affected family members followed, as most frequently found for *RHO* mutations, an autosomal dominant mode of inheritance. Only few mutations in genes of the phototransduction pathway may lead to CSNB, whereas the majority of them cause more severe phenotypes such as RP^24^. Mutations in the *RHO* gene are known to be associated with two different phenotypes: the Riggs-type CSNB and RP^3^,^4^,^5^,^35^. Patients affected with the Riggs-type CSNB present a lifelong mild phenotype, consisting of night blindness and normal visual acuity, lacking any signs of a high myopia and nystagmus^4^. The phenotype of RP first resembles night blindness but progresses towards a degeneration of rod and subsequently cone photoreceptors and may lead to complete blindness. It has been shown that various mutations in *RHO* are harmful to rod photoreceptors. The toxic effects of *RHO* mutations may lead to a formation of intracellular protein aggregates, affect the intracellular transport, or alter the structure of rod photoreceptors^14^. These cellular changes are manifested by decreased retinal outer layer thickness and retinal lesions or pigment deposits in the fundus^2^.

We found both, the Riggs-type CSNB and RP phenotypes within members of the same family. We identified a *RHO* mutation which co-segregates with both phenotypes and is dominantly inherited in the family described herein. A dominant form of CSNB in combination with mutations in RHO (specifically localizing to rod photoreceptors) supports the diagnosis of Riggs-type CSNB. A bifid wave form of the photopic 30Hz flicker ERG recordings was published to be a sign of an incomplete CSNB^13^. Interestingly, we found the double peak in the flicker responses in the family member likely affected with Riggs-type CSNB. To the best of our knowledge this is the first time that this phenomenon was observed in Riggs-type CSNB patients. We speculate that the combination of Riggs-type ERG and the double peak 30 Hz flicker recordings might be specific for the mutation in the RHO counterion. However, the analysis of additional families with this mutation will be required to clarify this notion.

To date, it is unclear why the same RHO mutation is associated with two phenotypes. Possible explanations for this observation might include modifier variants in the genome of the index patient, live circumstances or stoichiometric factors that tipped the balance towards one or the other clinical phenotype. Sieving *et al*. 1995 presented that the p.G90D mutation in human *RHO* leads to an early onset, extensive night blindness without obvious signs of a progressive retinal degeneration. These findings were afterwards confirmed on mouse rods *in vivo*^36^. Further investigations in transgenic mice revealed that an overexpression of RHO protein, whether wild type or p.G90D transgenes, increases the severity of a retinal degeneration and leads to a disruption of photoreceptor morphology^37^. Accordingly, we speculate that two different phenotypes, RP in the index patient and Riggs-type CSNB in the clinically examined patient, may occur due to a different amount of *RHO* mutant allele (p.E113K). Since data available from family members other than the index showed exclusively night blindness without any signs that point towards a progressive retinal degenerative process, we speculate that the RHO mutation p.E113K might cause Riggs-type CSNB also in other patients. This notion is supported by the oldest family member, who now is 82 years old and did not show any signs supporting a progressive nature of her disease phenotype. Nevertheless, it cannot be completely excluded that a protective factor was lost in the RP affected index. Additional factors such as epigenetic modifications may play an important role in causing the RP phenotype in the index patient. For example, an increased DNA-methylation was found in dying photoreceptors from rodent models for RP. This finding emphasizes an important role of the DNA modification in photoreceptors’ degeneration^38^. Whether epigenetic regulations or additional factors might have led to the RP phenotype in the index patient remains unclear.

Rhodopsin belongs to a group of heptahelical membrane proteins and locates to the outer segment of the retinal photoreceptors^20^,^34^,^39^. It is built up by an opsin protein that covalently binds to the 11-cis chromophore at amino acid lysine 296. The chromophore is bound by a protonated Schiff base linkage. The Schiff base amino acid p.K296 was identified in the seventh transmembrane helix of RHO, while the counterion p.E113 locates to the third transmembrane helix. The counterion stabilizes the protonated Schiff base linkage^34^. The salt bridge between p.E113 and p.K296 is important to form a functional RHO^20^,^25^. The light-induced isomerisation of the 11-cis chromophore leads to an all-trans form and initiates the generation of a cascade of photointermediates of RHO. These conformation changes require an interaction of all seven RHO helices with the chromophore. The key photoproduct, metarhodopsin II, forms transiently and is able to activate a G-protein-mediated transduction cascade, called the phototransduction cascade^40^. According to the counterion switch model presented by Yan *et al*. 2003, the Schiff base in the metarhodopsin II requires an interaction of the counterion p.E181 of the Schiff base in the metarhodospin I with the p.E113^41^. The relative contribution of p.E113 and p.E181 to the stabilization of the Schiff base proton in different intermediates of RHO seems not yet completely understood^42^. The hydrolysis of the Schiff base linkage is thought to trigger further conformational changes resulting in a dissociation of the all-trans-retinal. Consequently, opsin remains inactive until another cycle of activation^25^.

Due to its predominant role in mediating light conversion, the Schiff base counterion p.E113 became an interesting field of research. Different *in-vitro* studies have been performed in order to better understand the role of the counterion in RHO function^20^,^43^. Three different mutants in the bovine RHO were studied by Sakmar *et al*. 1989: p.E113Q, p.E113D, and p.E113 K. The authors observed that the mutants, p.E113D and p.E113Q, gave a slightly red- and a prominent blue-shifted pigment, respectively. However, the p.E113 K mutant was unable to bind 11-cis-retinal to stabilize the chromophore within RHO. They revealed that the Schiff base protonation, essential for a functional RHO, cannot occur with the p.E113 K mutation.

It has also been described that the counterion plays a role in suppressing the constitutive activation of opsin. Several constitutively active mutants of RHO, including p.E113Q and p.K296A/E/G^25^ cause a damage of the salt bridge between p.E113 and p.L296^35^,^44^. Additionally, mutants located in the close vicinity to the counterion modulate the efficiency of forming the Schiff base with the chromophore. Some of them (e.g. p.G90D and p.T94D) strikingly reduced whereas others (e.g. A292E, and T94I) increased a speed of the Schiff base formation. The counterion is thought to be a “catalytic base” involved in the rate of the 11-cis chromophore binding^45^. Moreover, an involvement in the spectral tuning^46^ and in a photoisomerization efficiency^47^, as well as a regulation of the lifetime of metarhodospin II were associated with the p.E113 counterion properties^20^,^42^,^47^. Night blindness-associated mutations often cause RHO to constitutively activate the phototransduction cascade at a low level. All 4 known amino acids exchanges leading to autosomal dominant CSNB locate to similar positions in the 3D model of RHO (see Supplementary Fig. S7). This supports the notion that these mutations lead to de-stabilization of the chromophore and therefore cause a constitutive activation of RHO. This provides a disease mechanism that is likely to be relevant to rod photoreceptor-mediated CSNB due to a reduced sensitivity to light stimuli.

In conclusion, this report is, to the best of our knowledge, the first description of a patient-derived mutation directly affecting the Schiff base counterion p.E113. Clinically, the mutation can be associated with both, a moderately progressive form of RP or a rare Riggs-type CSNB. We found both of these phenotypes in the same family.

## Additional Information

**How to cite this article**: Reiff, C. *et al*. The mutation p.E113K in the Schiff base counterion of rhodopsin is associated with two distinct retinal phenotypes within the same family. *Sci. Rep*. **6**, 36208; doi: 10.1038/srep36208 (2016).

**Publisher’s note:** Springer Nature remains neutral with regard to jurisdictional claims in published maps and institutional affiliations.

## Supplementary Material

Supplementary Information

## References

[b1] Veleri S. . Biology and therapy of inherited retinal degenerative disease: insights from mouse models. Disease models & mechanisms 8, 109–129 (2015). 25650393 10.1242/dmm.017913 PMC4314777

[b2] Berger W., Kloeckener-Gruissem B. & Neidhardt J. The molecular basis of human retinal and vitreoretinal diseases. Progress in retinal and eye research 29, 335–375 (2010). 20362068 10.1016/j.preteyeres.2010.03.004

[b3] Neidhardt J., Barthelmes D., Farahmand F., Fleischhauer J. C. & Berger W. Different amino acid substitutions at the same position in rhodopsin lead to distinct phenotypes. Investigative ophthalmology & visual science 47, 1630–1635 (2006). 16565402 10.1167/iovs.05-1317

[b4] Zeitz C., Robson A. G. & Audo I. Congenital stationary night blindness: an analysis and update of genotype-phenotype correlations and pathogenic mechanisms. Progress in retinal and eye research 45, 58–110 (2015). 25307992 10.1016/j.preteyeres.2014.09.001

[b5] Ferrari S. . Retinitis pigmentosa: genes and disease mechanisms. Current genomics 12, 238–249 (2011). 22131869 10.2174/138920211795860107 PMC3131731

[b6] Hamel C. Retinitis pigmentosa. Orphanet journal of rare diseases 1, 40 (2006). 17032466 10.1186/1750-1172-1-40 PMC1621055

[b7] Berger A. . Repair of rhodopsin mRNA by spliceosome-mediated RNA trans-splicing: a new approach for autosomal dominant retinitis pigmentosa. Molecular therapy: the journal of the American Society of Gene Therapy 23, 918–930 (2015). 25619725 10.1038/mt.2015.11 PMC4427870

[b8] Berson E. L. Retinitis pigmentosa. The Friedenwald Lecture. Investigative ophthalmology & visual science 34, 1659–1676 (1993). 8473105

[b9] Riggs L. A. Electroretinography in cases of night blindness. American journal of ophthalmology 38, 70–78 (1954). 13180620 10.1016/0002-9394(54)90011-2

[b10] Schubert G. & Bornschein H. Analysis of the human electroretinogram. Ophthalmologica Journal international d’ophtalmologie International journal of ophthalmology Zeitschrift fur Augenheilkunde 123, 396–413 (1952). 10.1159/000301211 14957416

[b11] Riazuddin S. A. . A mutation in SLC24A1 implicated in autosomal-recessive congenital stationary night blindness. American journal of human genetics 87, 523–531 (2010). 20850105 10.1016/j.ajhg.2010.08.013 PMC2948789

[b12] Miyake Y., Yagasaki K., Horiguchi M., Kawase Y. & Kanda T. Congenital stationary night blindness with negative electroretinogram. A new classification. Archives of ophthalmology 104, 1013–1020 (1986). 3488053 10.1001/archopht.1986.01050190071042

[b13] Miyake Y., Horiguchi M., Ota I. & Shiroyama N. Characteristic ERG-flicker anomaly in incomplete congenital stationary night blindness. Investigative ophthalmology & visual science 28, 1816–1823 (1987). 3499417

[b14] Hartong D. T., Berson E. L. & Dryja T. P. Retinitis pigmentosa. Lancet 368, 1795–1809 (2006). 17113430 10.1016/S0140-6736(06)69740-7

[b15] Glockle N. . Panel-based next generation sequencing as a reliable and efficient technique to detect mutations in unselected patients with retinal dystrophies. European journal of human genetics: EJHG 22, 99–104 (2014). 23591405 10.1038/ejhg.2013.72 PMC3865404

[b16] Dryja T. P., Berson E. L., Rao V. R. & Oprian D. D. Heterozygous missense mutation in the rhodopsin gene as a cause of congenital stationary night blindness. Nature genetics 4, 280–283 (1993). 8358437 10.1038/ng0793-280

[b17] Rao V. R., Cohen G. B. & Oprian D. D. Rhodopsin Mutation G90d and a Molecular Mechanism for Congenital Night Blindness. Nature 367, 639–642 (1994). 8107847 10.1038/367639a0

[b18] al-Jandal N. . A novel mutation within the rhodopsin gene (Thr-94-Ile) causing autosomal dominant congenital stationary night blindness. Human mutation 13, 75–81 (1999). 9888392 10.1002/(SICI)1098-1004(1999)13:1<75::AID-HUMU9>3.0.CO;2-4

[b19] Zeitz C. . Identification and functional characterization of a novel rhodopsin mutation associated with autosomal dominant CSNB. Investigative ophthalmology & visual science 49, 4105–4114 (2008). 18487375 10.1167/iovs.08-1717

[b20] Sakmar T. P., Franke R. R. & Khorana H. G. Glutamic acid-113 serves as the retinylidene Schiff base counterion in bovine rhodopsin. Proceedings of the National Academy of Sciences of the United States of America 86, 8309–8313 (1989). 2573063 10.1073/pnas.86.21.8309 PMC298270

[b21] Stryer L. Cyclic GMP cascade of vision. Annual review of neuroscience 9, 87–119 (1986). 10.1146/annurev.ne.09.030186.000511 2423011

[b22] Sharon D. . Mutated alleles of the rod and cone Na-Ca + K-exchanger genes in patients with retinal diseases. Investigative ophthalmology & visual science 43, 1971–1979 (2002). 12037007

[b23] Tsang S. H. . Transgenic mice carrying the H258N mutation in the gene encoding the beta-subunit of phosphodiesterase-6 (PDE6B) provide a model for human congenital stationary night blindness. Human mutation 28, 243–254 (2007). 17044014 10.1002/humu.20425 PMC2753261

[b24] Zeitz C. Molecular genetics and protein function involved in nocturnal vision. Expert Rev Ophthalmol 2, 467–485 (2007).

[b25] Robinson P. R., Cohen G. B., Zhukovsky E. A. & Oprian D. D. Constitutively active mutants of rhodopsin. Neuron 9, 719–725 (1992). 1356370 10.1016/0896-6273(92)90034-b

[b26] McCulloch D. L. . ISCEV Standard for full-field clinical electroretinography (2015 update). Documenta ophthalmologica Advances in ophthalmology 130, 1–12 (2015). 10.1007/s10633-014-9473-7 25502644

[b27] Hood D. C. . ISCEV standard for clinical multifocal electroretinography (mfERG) (2011 edition). Documenta ophthalmologica Advances in ophthalmology 124, 1–13 (2012). 10.1007/s10633-011-9296-8 PMC4466109 22038576

[b28] Poloschek C. M. . ABCA4 and ROM1: implications for modification of the PRPH2-associated macular dystrophy phenotype. Investigative ophthalmology & visual science 51, 4253–4265 (2010). 20335603 10.1167/iovs.09-4655

[b29] Li H. & Durbin R. Fast and accurate short read alignment with Burrows-Wheeler transform. Bioinformatics 25, 1754–1760 (2009). 19451168 10.1093/bioinformatics/btp324 PMC2705234

[b30] Li H. . The Sequence Alignment/Map format and SAMtools. Bioinformatics 25, 2078–2079 (2009). 19505943 10.1093/bioinformatics/btp352 PMC2723002

[b31] McKenna A. . The Genome Analysis Toolkit: a MapReduce framework for analyzing next-generation DNA sequencing data. Genome research 20, 1297–1303 (2010). 20644199 10.1101/gr.107524.110 PMC2928508

[b32] Van der Auwera G. A. . From FastQ data to high confidence variant calls: the Genome Analysis Toolkit best practices pipeline. Current protocols in bioinformatics/editoral board, Andreas D Baxevanis 11, 11 10 11–11 10 33 (2013). 10.1002/0471250953.bi1110s43 PMC4243306 25431634

[b33] Untergasser A. . Primer3–new capabilities and interfaces. Nucleic acids research 40, e115 (2012). 22730293 10.1093/nar/gks596 PMC3424584

[b34] Khorana H. G. Rhodopsin, photoreceptor of the rod cell. An emerging pattern for structure and function. The Journal of biological chemistry 267, 1–4 (1992). 1730574

[b35] Rao V. R. & Oprian D. D. Activating mutations of rhodopsin and other G protein-coupled receptors. Annual review of biophysics and biomolecular structure 25, 287–314 (1996). 10.1146/annurev.bb.25.060196.001443 8800472

[b36] Sieving P. A. . Constitutive “light” adaptation in rods from G90D rhodopsin: a mechanism for human congenital nightblindness without rod cell loss. The Journal of neuroscience: the official journal of the Society for Neuroscience 21, 5449–5460 (2001). 11466416 10.1523/JNEUROSCI.21-15-05449.2001 PMC6762654

[b37] Naash M. I. . Retinal abnormalities associated with the G90D mutation in opsin. The Journal of comparative neurology 478, 149–163 (2004). 15349976 10.1002/cne.20283

[b38] Farinelli P. . DNA methylation and differential gene regulation in photoreceptor cell death. Cell death & disease 5, e1558 (2014). 25476906 10.1038/cddis.2014.512 PMC4649831

[b39] Ramos L. S., Chen M. H., Knox B. E. & Birge R. R. Regulation of photoactivation in vertebrate short wavelength visual pigments: protonation of the retinylidene Schiff base and a counterion switch. Biochemistry 46, 5330–5340 (2007). 17439245 10.1021/bi700138g

[b40] Wald G. Molecular basis of visual excitation. Science 162, 230–239 (1968). 4877437 10.1126/science.162.3850.230

[b41] Yan E. C. . Retinal counterion switch in the photoactivation of the G protein-coupled receptor rhodopsin. Proceedings of the National Academy of Sciences of the United States of America 100, 9262–9267 (2003). 12835420 10.1073/pnas.1531970100 PMC170906

[b42] Tsutsui K. & Shichida Y. Multiple functions of Schiff base counterion in rhodopsins. Photochemical & photobiological sciences: Official journal of the European Photochemistry Association and the European Society for Photobiology 9, 1426–1434 (2010). 10.1039/c0pp00134a 20842311

[b43] Sakmar T. P., Franke R. R. & Khorana H. G. The role of the retinylidene Schiff base counterion in rhodopsin in determining wavelength absorbance and Schiff base pKa. Proceedings of the National Academy of Sciences of the United States of America 88, 3079–3083 (1991). 2014228 10.1073/pnas.88.8.3079 PMC51388

[b44] Kim J. M. . Structural origins of constitutive activation in rhodopsin: Role of the K296/E113 salt bridge. Proceedings of the National Academy of Sciences of the United States of America 101, 12508–12513 (2004). 15306683 10.1073/pnas.0404519101 PMC515088

[b45] Gross A. K., Rao V. R. & Oprian D. D. Characterization of rhodopsin congenital night blindness mutant T94I. Biochemistry 42, 2009–2015 (2003). 12590588 10.1021/bi020613j

[b46] Janz J. M. & Farrens D. L. Assessing structural elements that influence Schiff base stability: mutants E113Q and D190N destabilize rhodopsin through different mechanisms. Vision research 43, 2991–3002 (2003). 14611935 10.1016/j.visres.2003.08.010

[b47] Tsutsui K. & Shichida Y. Photosensitivities of rhodopsin mutants with a displaced counterion. Biochemistry 49, 10089–10097 (2010). 21038858 10.1021/bi101020p

