# Supplementary material for: The mutation p.E113K in the Schiff base counterion of rhodopsin is associated with two distinct retinal phenotypes within the same family
Source: Sci Rep. 2016 Nov 4;6:36208. doi: 10.1038/srep36208 (PMC5095885; doi:10.1038/srep36208)
Supplement: Supplementary Information [file srep36208-s1.doc]

**Supplementary material**

**The mutation p.E113K in the Schiff base counterion of rhodopsin is associated with two distinct retinal phenotypes within the same family.**

Charlotte Reiff^1+^, Marta Owczarek-Lipska^2+^, Georg Spital^3^, Carsten Röger^2^, Hebke Hinz^2^, Christoph Jüschke^2^, Holger Thiele^4^, Janine Altmüller^4^, Peter Nürnberg^4^, Romain Da Costa^2^ and John Neidhardt^2*^

**
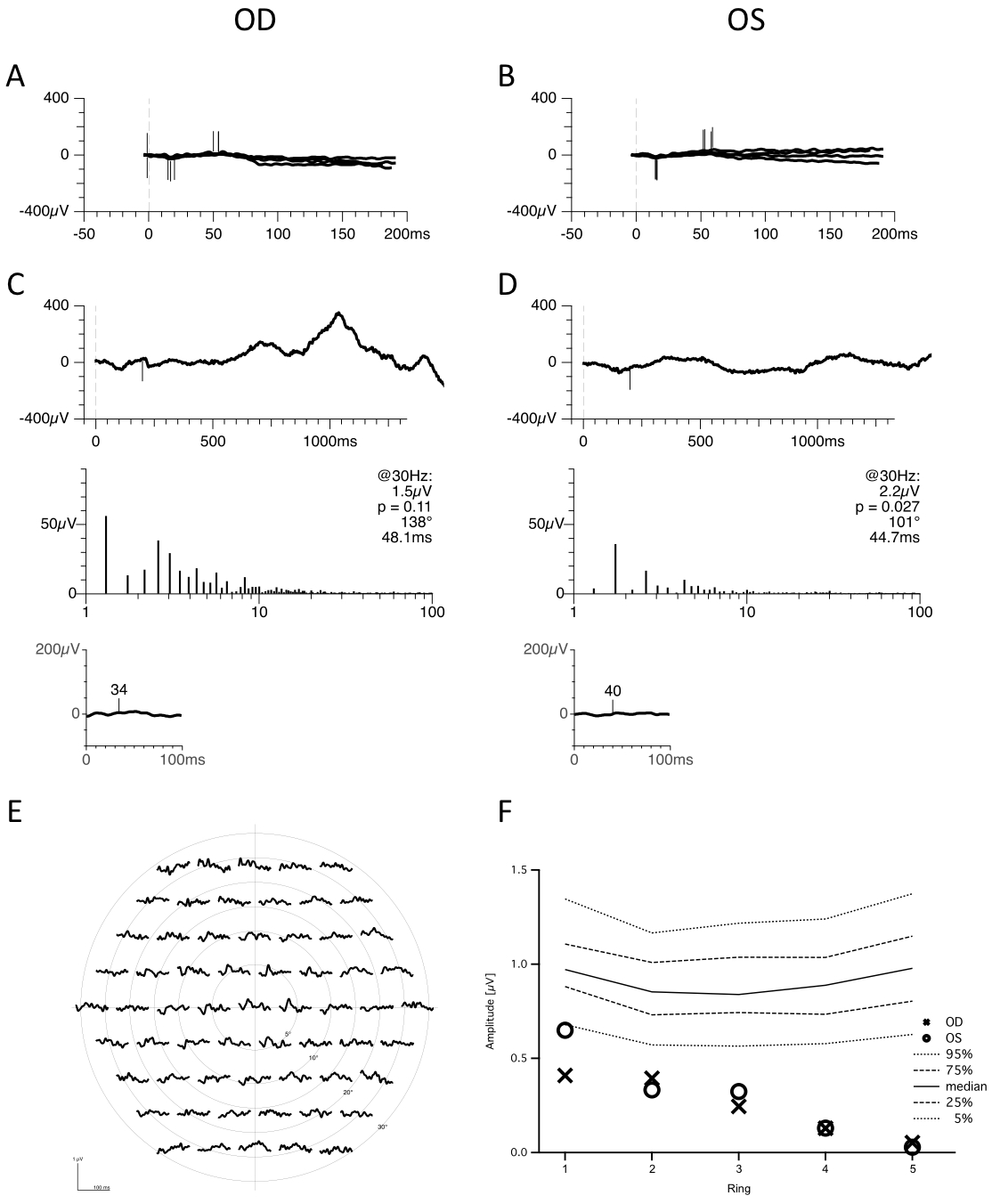
**

**Fig. S1. ERG (A – D) and mfERG (E – F) of the index patient.** OD: right eye, OS: left eye. A, B: dark-adapted 10 ERG shows pathologically reduced, but detectable and reproducible minor amplitudes in both eyes. C, D: discrete Fourier transform (middle row) of the flicker response (upper row) revealed minor significant cone responses of the left eye only. Lower row: waveform of the 30 Hz flicker response. E: trace array of the right eye (left eye not shown due to interocular symmetry): pathological central amplitude reduction and decreasing amplitudes with increasing eccentricity of the stimulus. F: ring averages of right eye (cross) and left eye (circle) are below the 5^th^ percentile and decrease with increasing eccentricity.


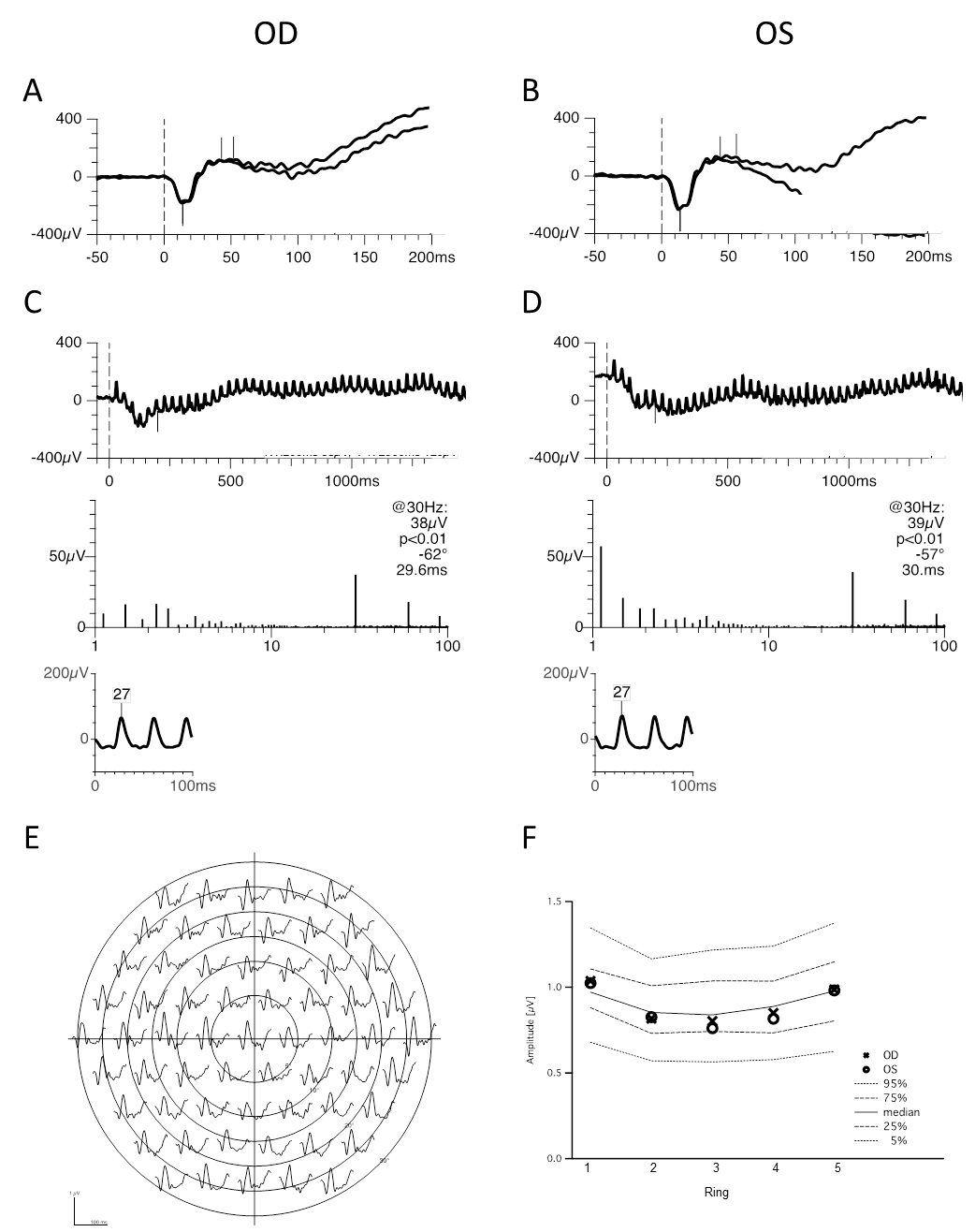


**Fig. S2. Normal ERG (A – D) and normal mfERG (E – F).** OD: right eye, OS: left eye. A, B: dark-adapted 10 ERG. C, D: discrete Fourier transform (middle row) of the flicker response (upper row). Lower row: waveform of the 30 Hz flicker response. E: trace array of the right eye. F: ring averages of right eye (cross) and left eye (circle).

**
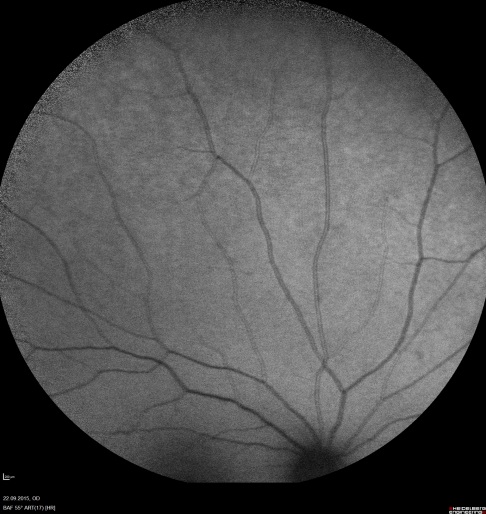

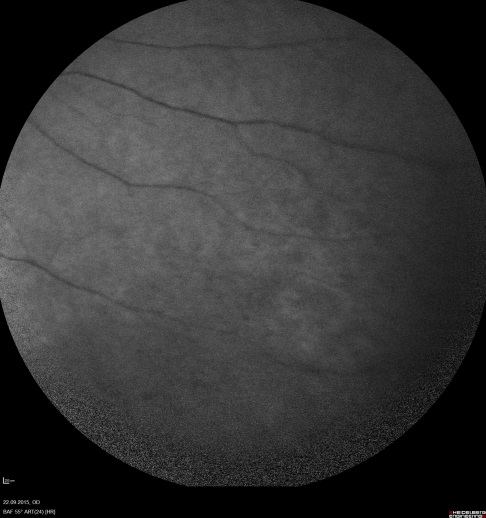
**

**OD**

**A.**

**B.**

**
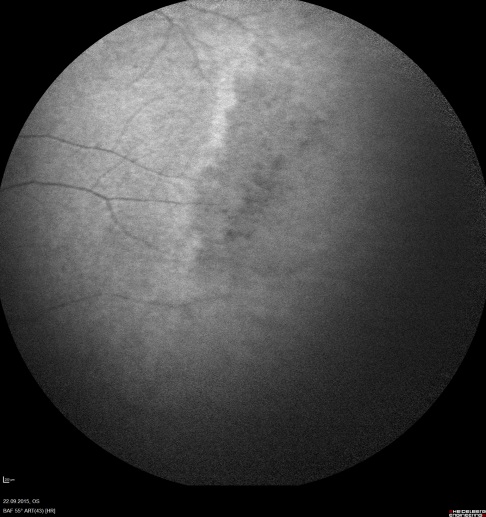

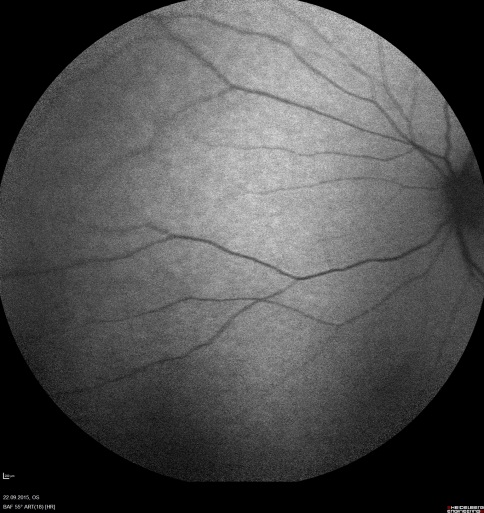

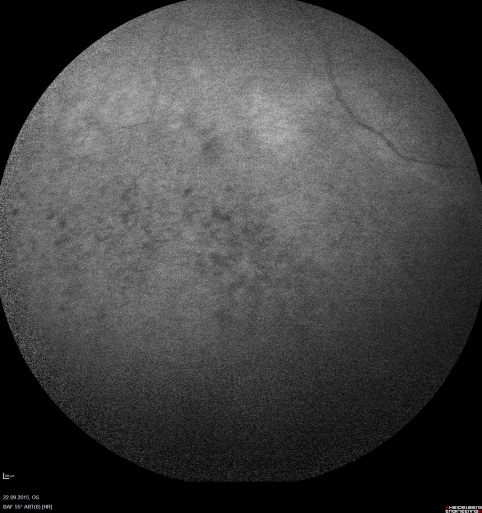
**

**OS**

**C.**

**D.**

**E.**

**Fig. S3. Clinical characterization of the patient II:3 diagnosed with Riggs-type CSNB.** FAF of the right eye (OD) and of the left eye (OS) are shown from retinal areas that are unaltered (A, C) and from areas that present with peripheral alterations of unclear origin and clinical significance (B, D, E).

A.


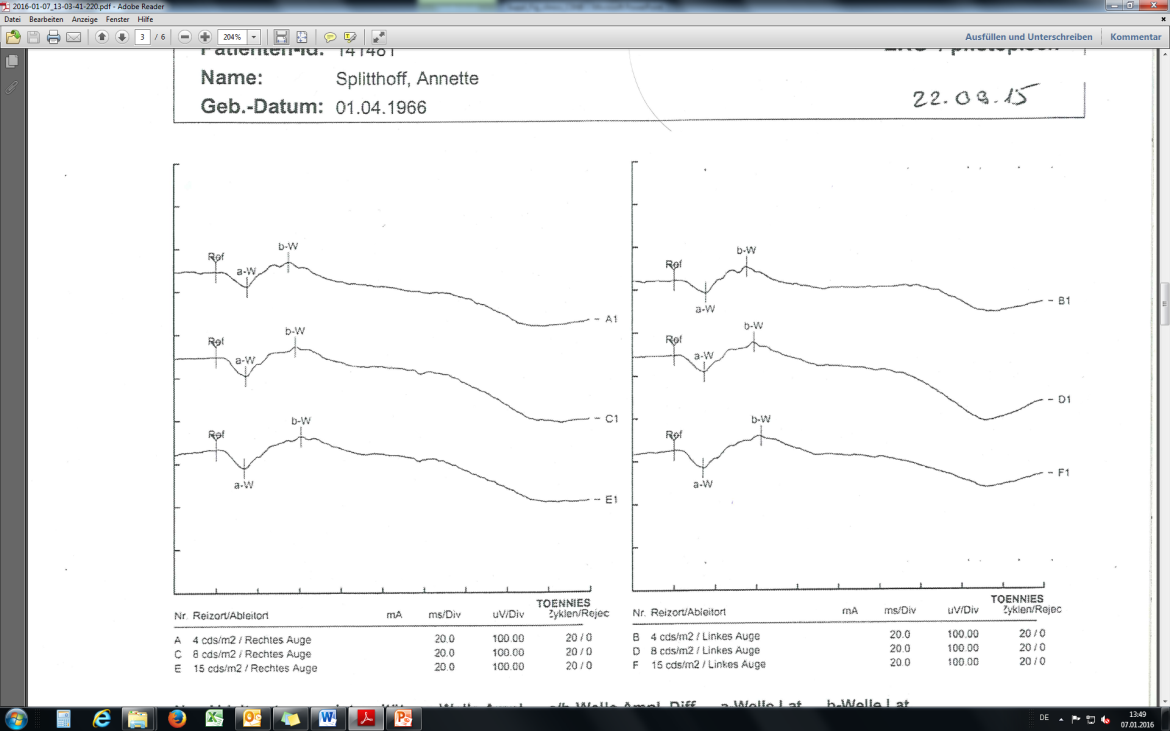


B.

C.

**OD**

**OS**

photopic


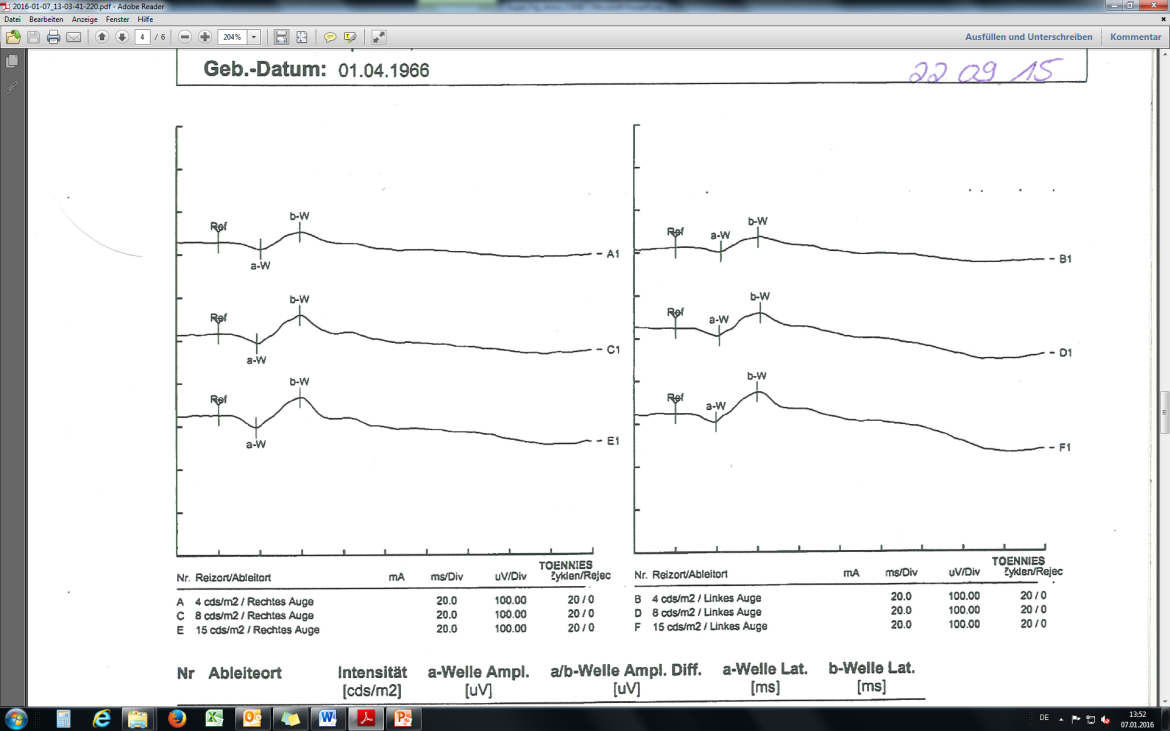

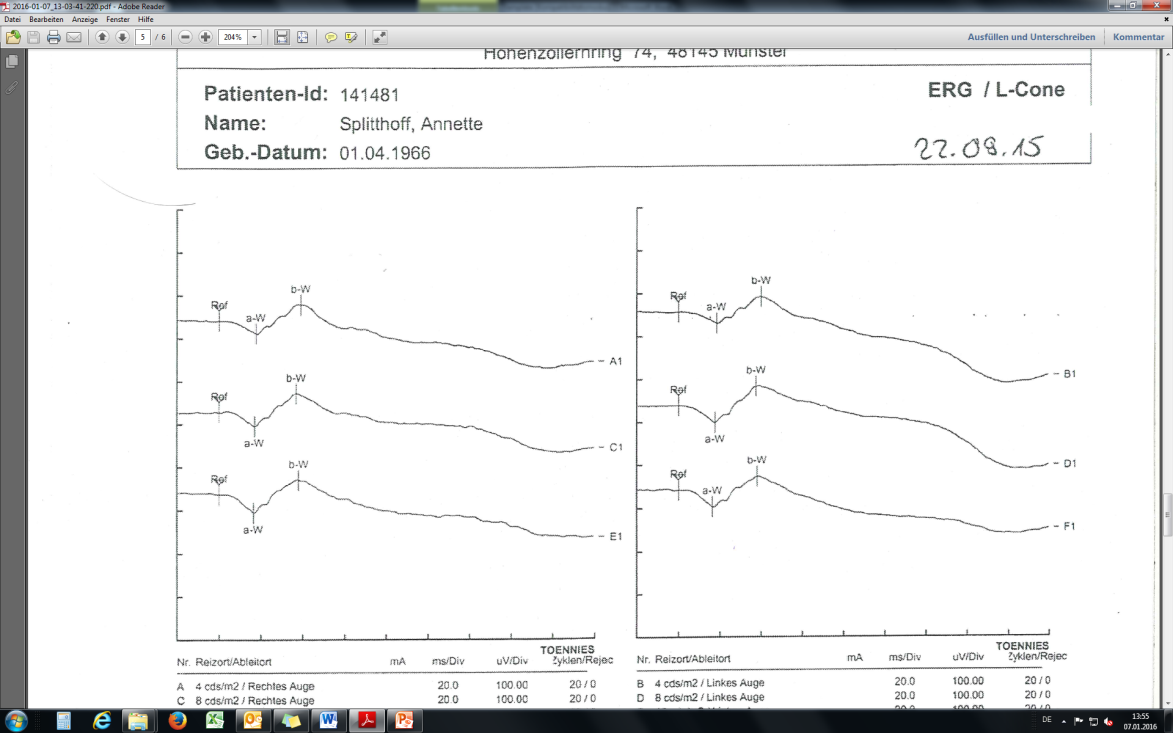


S-cone

L-cone

**Fig. S4. ERGs (A-C) of the patient II:3 diagnosed with Riggs-type CSNB.** OD represents measurements in the right eye, where OS corresponds to the left eye. The ERGs were measured in both eyes with intensity series of 4 cds/m2 (A1, B1), 8 cds/m2 (C1, D1), and 15 cds/m2 (E1, F1). Units on the x and y axes show 20 ms and 100 µV per division mark, respectively.

A.

B.

scotopic

flicker

**OD**

**OS**


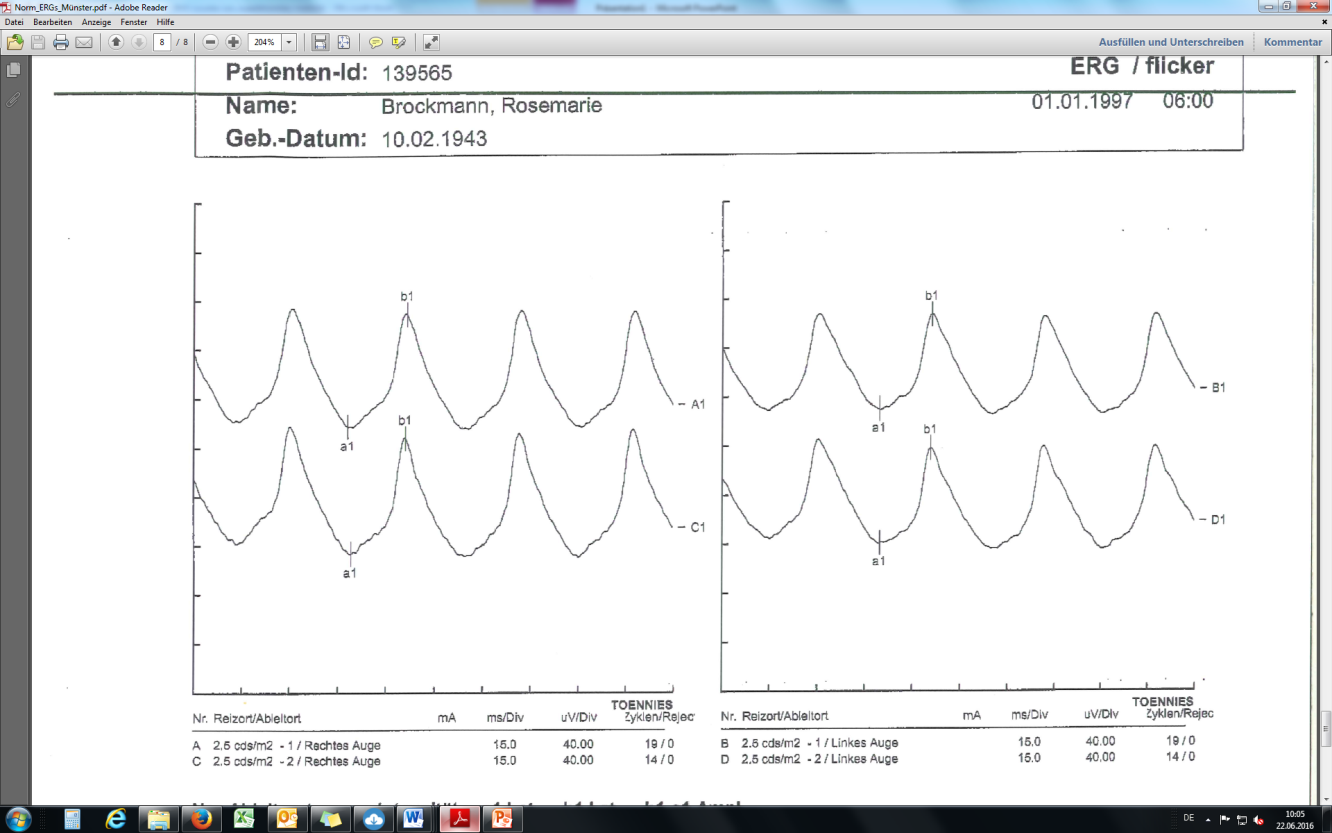

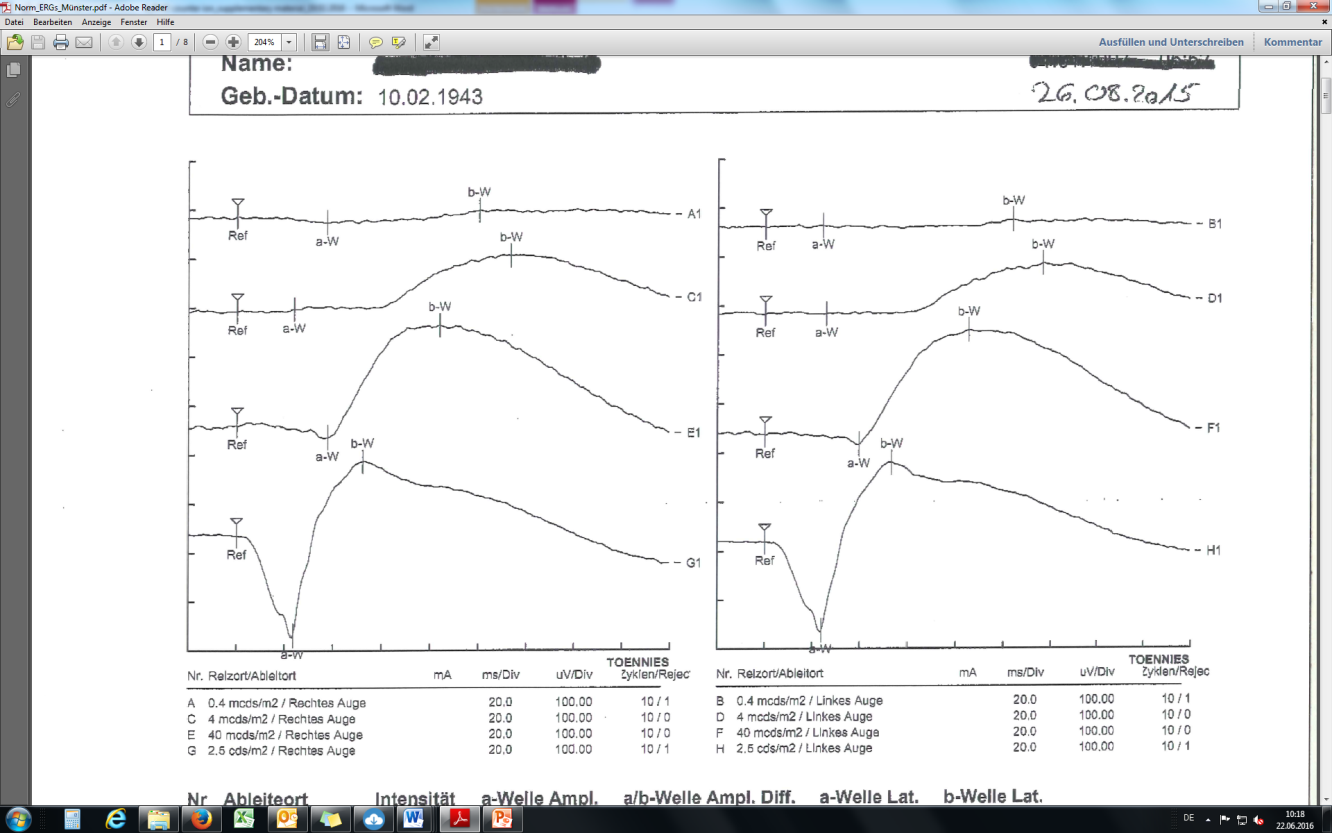


**Fig. S5. Normal ERGs of a healthy control individual.** OD corresponds to the right eye and OS represents the left eye. A: The scotopic ERGs were measured in both eyes with four intensities: 0.4 mcds/m2 (A1, B1), 4 mcds/m2 (C1, D1), 40 mcds/m2 (E1, F1), and 2500 mcds/m2 (G1, H1). Units on the x and y axes show 20 ms and 100 µV per division mark, respectively. B: The flicker ERGs with the 2.5 cds/m2 intensity are shown in OD (A1, C1) and in OS (B1, D1). Units on the zoomed in x and y axes of the 30 Hz flicker show 15 ms and 40 µV per division mark, respectively.

A.

B.

C.

S-cone

L-cone

photopic


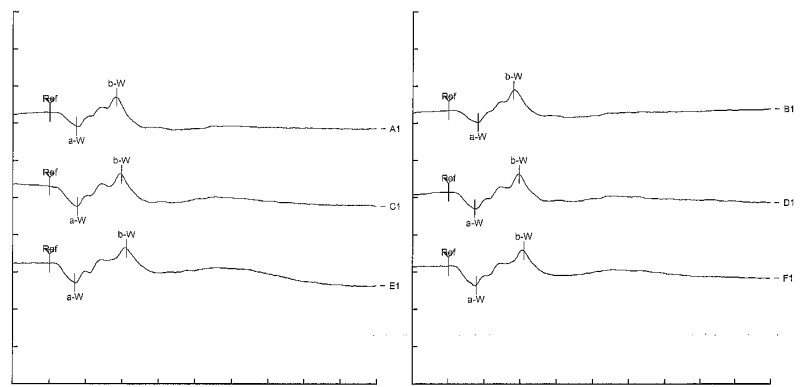

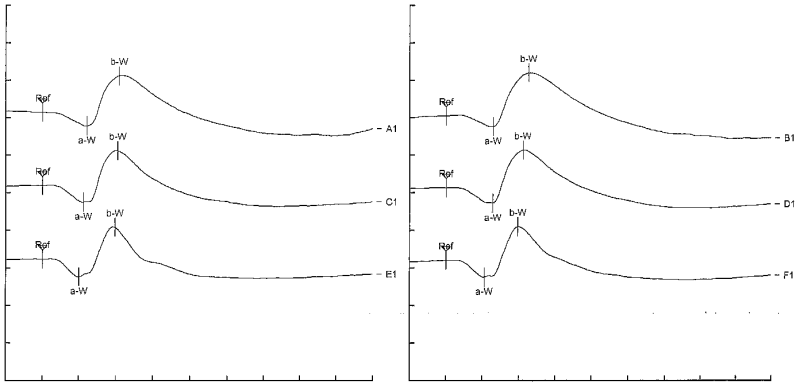

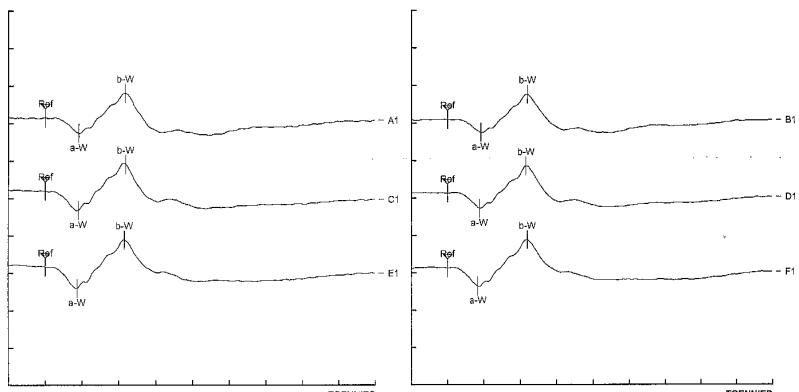


**OD**

**OS**

**Fig. S6. Normal ERGs (A-C) of a healthy control individual.** OD: the right eye, OS: the left eye. The ERGs were measured in both eyes with intensity series of 4 cds/m2 (A1, B1), 8 cds/m2 (C1, D1), and 15 cds/m2 (E1, F1). Units on the x and y axes show 20 ms and 100 µV per division mark, respectively.

**
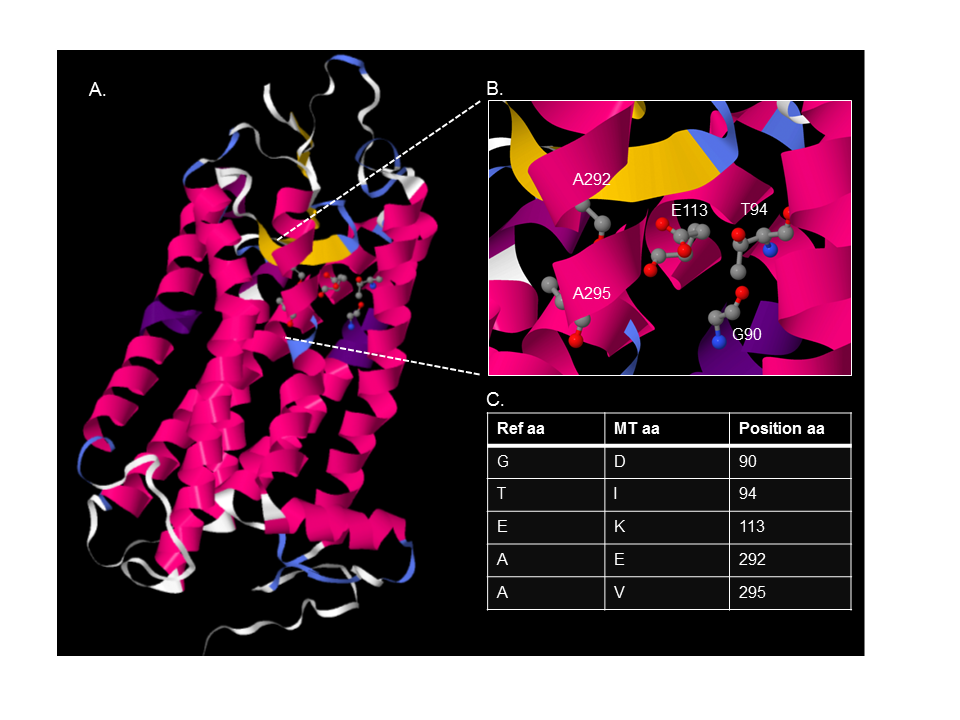
**

**Fig S7. A 3D-model of the RHO protein presenting constitutively active mutations associated with autosomal dominant CSNB.** A: A complete structure of the RHO protein was remodelled with SWISS-MODEL (<http://www.expasy.ch/swissmod/SWISS-MODEL.html>) and Jmol software (<http://jmol.sourceforge.net/download/>) using the human RHO protein sequence (NP_000530.1). Four amino acids (aa) residues (G90, T94, A292, and A295) known to be mutated in autosomal dominant CSNB-cases as well as the counterion position p.E113 are presented by spheres. B: A zoom into the aforementioned aa residues. C: Table summarizing four known RHO mutations leading to autosomal dominant CSNB in addition to the newly identified p.E113K mutation. Ref aa: reference aa, MT aa: mutated aa.

**Table S1. Genetic variants detected by whole exome sequencing in the RP-affected index patient.**

| **Type of sequence change** | **Number of variants** |
| --- | --- |
| Non-synonymous, coding | 347 |
| Synonymous, coding | 3 |
| Nonsense | 9 |
| Insertion/deletion | 7 |
| Splice site | 6 |

**Table S2. Allele frequency and average heterozygosity of three likely non-pathogenic sequence variants in *RHO*.**

| **Location** | **Genetic variant** | **dbSNP** | **Allele frequencies (%)**  **WT MT** | | **Average heterozygosity*** |
| --- | --- | --- | --- | --- | --- |
| 5‘UTR | c.-26A>G ^§^ | rs7984 | A (61.016) | G (38.984) | 0.498 +/- 0.029 |
| Intron 3 | c.696+4C>T ^§^ | rs56340615 | C (91.222) | T (8.778) | 0.148 +/- 0.228 |
| 3‘UTR | c.*46C>A ^§^ | rs2071093 | C (92.301) | A (7.699) | 0.140 +/- 0.225 |

* from UCSC Genome Browser, human genome assembly (GRCh37/hg19).

^§^ identified in all family members
